# Lawton’s Instrumental Activities of Daily Living for Greek-Speaking Adults with Cognitive Impairment: A Psychometric Evaluation Study with Additional Receiver Operating Characteristic Curve Analysis

**DOI:** 10.3390/brainsci13071093

**Published:** 2023-07-19

**Authors:** Dionysios Tafiadis, Vassiliki Siafaka, Louiza Voniati, Alexandra Prentza, Angelos Papadopoulos, Nafsika Ziavra, Spyridon Konitsiotis

**Affiliations:** 1 Department of Speech & Language Therapy, School of Health Sciences, University of Ioannina, GR45500 Ioannina, Greece; siafaka@uoi.gr (V.S.); nziavra@uoi.gr (N.Z.); 2 Department of Health Sciences, Speech and Language Therapy, European University Cyprus, 22006 Nicosia, Cyprus; l.voniati@euc.ac.cy; 3 Department of Linguistics, School of Philology, Faculty of Philosophy, University of Ioannina, GR45500 Ioannina, Greece; aprentza@uoi.gr; 4 Department of Medicine, School of Health Sciences, University of Ioannina, GR45500 Ioannina, Greece; angelospapadopoulos@gmail.com (A.P.); skonitso@gmail.com (S.K.)

**Keywords:** Alzheimer’s disease, dementia, IADL, Parkinson’s disease, ROC curve, quality of life

## Abstract

One of the components of a dementia diagnosis is the assessment of functional abilities. These abilities are measured via screeners, such as the Instrumental Activities of Daily Living (IADL) scale. The IADL scale is a valid tool that has been adapted in many languages. This study aimed to provide a cut-off point and validate the Greek version of the IADL scale in populations with cognitive impairment. IADL data were collected from 132 individuals: 24 PD patients, 24 Parkinson’s disease dementia (PDD) patients, and 24 AD patients. The remaining 60 participants were cognitive healthy adults (CHAs). The CHA group and the PD group served as the cognitively unimpaired group (CUG), while the PDD and AD groups served as the cognitively impaired group (CIG). Additionally, the MMSE, the AMTS, the Clock Drawing Test CDT, the Arizona Battery for Communication Disorders of Dementia (ABCD), the NPI, and the GDS-15 were administered to the participants. Statistically significant differences in the IADL scores were exhibited between all subgroups. The IADL scale showed high internal consistency (Cronbach’s alpha = 0.890). A threshold equal to 6.00 (AUC = 0.888, *p* < 0.001) was estimated between the CUG and the CIG. Significant positive correlations were observed between IADL and MMSE (r = 0.764, *p* < 0.001), IADL and AMTS (r = 0.724, *p* < 0.001), IADL and ABCD (r = 0.702, *p* < 0.001), and IADL and CDT (r = 0.627, *p* < 0.001) results. Given the obtained results, the IADL scale is a valid tool for clinical use with high reliability and sensitivity. Also, the IADL scale is a valuable instrument for screening functional abilities associated with cognitive impairment.

## 1. Introduction

The DSM-5 criteria now include an updated definition of dementia. Major neurocognitive disorder (MND) has replaced the former name of dementia [1,2]. However, this article will refer to it as dementia due to the widespread use of the term in medical literature and society. Dementia is a progressive brain disease that alters cognitive function beyond what might be expected from normal aging. This alteration is expressed as cognitive difficulties, which are the core characteristic feature of dementia. These deficits have a negative impact on a person’s functional capacity [1,2] and their daily living [1,2]. This negative impact varies according to the course of the disease [2]. Furthermore, the loss of a person’s functional capacity due to dementia also burdens financially the family budget and the world’s health system [3]. As indicated in the literature, reduced functional levels (instrumental activities of daily living) and chronic diseases in older adults may be directly or indirectly related to their quality of life [4,5].

Detecting signs and symptoms at the early stages of the disease with screening assessments is paramount in implementing appropriate interventions [2]. In order to diagnose dementia, a full patient history must be obtained, along with an evaluation of their cognitive impairment as well as an evaluation of the level of functional capacity in their daily activities [2]. The National Institute on Aging and Alzheimer’s Association (NIA–AA) [6] suggest criteria and assessment tools (screening and battery tests) that can be used in the characterization of cognitive impairment, whereas changes in everyday activities have been suggested as a criterion for dementia [6,7,8]. Many screening tools have been developed in the literature to assess the loss of functional capacity and problems arising in the daily living of a person who faces cognitive problems [2]. These screeners usually have a questionnaire or a caregiver’s interview that leads to a score that reflects the quality of daily living of a patient with cognitive impairment and/or dementia [2,9]. Some of the most widely used questionnaires are the Alzheimer’s Disease Cooperative Study–Activities of Daily Living Scale (ADCS–ADL) [9], the Activity of Daily Living Prevention Instrument (ADL-PI) [10], the Bayer Activities of Daily Living Scale (B-ADL) [11], and the Instrumental Activities of Daily Living (IADL) scale [12].

The IADL scale is an instrument that assesses independent living skills in the community setting [12]. These skills are considered more complex than the basic activities of daily living, as measured using the Katz index of ADLs [13,14]. The instrument is useful for identifying how a person is functioning and for recognizing the improvement or deterioration over time. There are eight domains of functions measured with Lawton’s IADL scale: “ability to use the telephone”, “shopping”, “food preparation”, “housekeeping”, “laundry”, “mode of transportation”, “responsibility for own medications”, and “ability to handle finances” [12]. The total score ranges from 0 (low functioning, dependent) to 8 (high functioning, independent).

The IADL scale is broadly used as a screening tool in patients in the early stages of dementia [15] and research [15,16]. Furthermore, Lawton’s IADL scale has been used as a model to create the new IADL instrument [17] for populations with cognitive impairment [16] and has been translated into many languages [4,16,18,19,20,21,22,23,24], including Greek [25,26]. Specifically, in their preliminary report, Theotoka et al. (2007) [25] translated and validated the IADL scale only for patients with Alzheimer’s disease, while Mystakidou et al. (2013) translated and validated the IADL scale for patients with advanced cancer [26].

This study aims to assess the psychometric properties of the Greek version of the IADL scale for populations with cognitive impairment due to Alzheimer’s and Parkinson’s disease. Additionally, this study provides cut-off points for the IADL total score by conducting a receiver operating characteristic (ROC) analysis. We hypothesize that Lawton’s IADL scale in the Greek language will: (a) have the same psychometric properties as reported in other studies and (b) have a discriminatory validity between individuals with or without cognitive impairment and therefore can be used in daily clinical practice.

## 2. Materials and Methods

### 2.1. Participants

In this study, 132 participants were enrolled. Of these, 60 were the informants of participants, recruited from the National Open Care Centre for the Elderly, an institution that was founded in 1984, and their goal is to protect the social rights of the elderly in Greece. These participants were in good cognitive health (cognitive healthy adult (CHA) group). The remaining 72 participants were the caregivers of 24 patients with Parkinson’s disease (PD) without cognitive impairment, 24 patients with PDD, and 24 patients with Alzheimer’s disease (AD). The last two groups, PDD and AD patients, formed the cognitively impaired group (CIG).

In contrast, the cognitively unimpaired group (CUG) consisted of the CHA group and PD patients without cognitive impairment. The caregivers of all patients were recruited from a neurological outpatient clinic of the University Hospital of Ioannina, Epirus, Greece. All patients (PD, PDD, and AD) were recruited from the same clinic. All participant subgroups were monolingual Greek speakers and matched in age, educational background, and gender. Before enrollment, all participants and caregivers were informed of this study and signed a written consent form.

All PD and AD patients received a formal diagnosis from a neurologist specializing in neurocognitive disorders. The diagnosis was based on neurological examination, medical history, and magnetic resonance imaging (MRI) [7,27,28]. The staging of PD patients was determined according to the Hoehn–Yahr staging system [29,30].

The PD and PDD patients’ cognitive status was estimated based on the Movement Disorders Society (MDS) task force criteria [27,28,29,30] and the clinical diagnostic criteria suggested by Emre et al. (2004) and Emre (2007) [31,32]. NIA–AA guidelines were followed to determine the level of cognitive impairment in AD patients [7,8]. Participants with a former history of other neurological impairment and prior cognitive deficits and/or psychiatric disorders were excluded from the study.

Additionally, for the exclusion of participants who had a history of psychiatric disorders and/or neuropsychiatric symptoms, the validated Greek version of the Geriatric Depression Scale-15 (GDS-15) [33] and the Greek version of Neuropsychiatric Inventory (NPI) [34] were administrated. The GDS-15 assesses depression in geriatric patients using 15 items in a closed format of “yes” or “no” questions [35]. In this study, a threshold set at 7, indicating the possible presence of depression according to the GDS-15, was used [33]. Participants with a GDS-15 score under 7 were considered eligible for the patient group and included in this study [33]. The NPI is a scale that assesses dementia-related behavioral problems [34]. It examines 12 subdomains of behavioral functioning: delusions, hallucinations, agitation/aggression, dysphoria, anxiety, euphoria, apathy, disinhibition, irritability/lability, aberrant motor activity, nighttime behavioral disturbances, and appetite and eating abnormalities. The frequency and severity of the symptoms are evaluated, and a total NPI score is calculated by adding the scores of the subdomains. Finally, the PD patients included in the CUG had the same years of disease duration, education, and marital status as the AD and PDD patients (see Table 1).

The authors assert that all procedures contributing to this work complied with the ethical standards of the relevant national and institutional committees on human experimentation and with the Declaration of Helsinki of 1975, as revised in 2008. All procedures involving human subjects/patients were approved by the Department of Medicine, School of Health Sciences, University of Ioannina (reference no.: 658α).

### 2.2. Data Collection and Instruments

The Greek version of the IADL scale was administered to all participants (the CHA group, the PD group, and the informants of the patients in the CIG), and Cronbach’s alpha coefficient was reported at 0.840 [25]. The IADL scale evaluates the level of functional abilities of a person with cognitive impairment via its eight domains [12]. The eight items are scored with 0 or 1 point according to a person’s ability on what they can and cannot do in their daily life [12].

Women are scored on all 8 areas of function; historically, for men, the areas of food preparation, housekeeping, and laundering are excluded to avoid gender bias. A summary score ranges from 0 (low functioning, dependent) to 8 (high functioning, independent) for women and from 0 to 5 for men [12]. In order to assess the cognitive status of the study’s participants, the following instruments were administered: the MMSE and the AMTS.

### 2.3. Mini Mental State Examination (MMSE)

The MMSE [36] is suggested by the MDS task force [28] and the NIA–AA [7,8] as an instrument for assessing cognitive impairment in PD and AD patients, respectively. It is a 30-point questionnaire commonly used to screen for dementia and to follow the course of cognitive changes over time. A threshold score of <24 was used to form the groups in this study using the Greek version of the MMSE [37].

### 2.4. Abbreviated Mental Test Score (AMTS)

The AMTS [38] is a 10-item screening test for detecting dementia in geriatric populations. The Greek version of the AMTS was used for categorizing patients with or without cognitive impairment in this study using a threshold of <6.5 [39].

The visuospatial and communication abilities of each participant were assessed using the following instruments: the ABCD and the Tuokko CDT.

### 2.5. Arizona Battery for Communication Disorders of Dementia (ABCD)

The ABCD is a battery test that consists of five constructs that assess: (a) M mental status, (b) episodic memory, (c) language expression, (d) language comprehension, and (e) visuospatial construction [40]. This test can categorize individuals with the possibility of developing cognitive impairment into four diagnostic categories: (i) PD without dementia, (ii) PDD, (iii) mild AD, and (iv) moderate AD. This study used the preliminary Greek version of the ABCD to classify patients into the categories previously mentioned [41,42].

### 2.6. The Tuokko Version of the Clock Drawing Test (CDT)

The Tuokko Clock Drawing Test scoring system [43] was administered to all participants. The Tuokko CDT quantifies the visuospatial abilities of patients according to the severity of their cognitive status [43]. All participants were scored according to the Greek version of the Tuokko CDT and categorized as individuals with or without cognitive problems using a cut-off point of <4 [44].

### 2.7. Statistical Analysis

The variables’ distribution was evaluated with Kolmogorov–Smirnov and Shapiro–Wilk tests. All variables were expressed with means (M) and standard deviations (SD). Student’s t-test was used for comparisons of the IADL total mean score and its 8 domain mean scores between the CHA group and the CIG. Additionally, one-way ANOVA was conducted for between-group comparisons of the IADL total mean score and its 8 domain mean scores. Cut-off values for the IADL total score were estimated through a receiver operating characteristic (ROC) curve analysis.

The internal consistency of the re-evaluated Greek version of the IADL scale was measured using Cronbach’s alpha coefficient and the split-half reliability coefficient technique. A value greater than 0.8 estimates “good” internal consistency and greater than 0.9 an “excellent” one. Furthermore, the Pearson correlation between the total scores of the Greek versions of the ABCD, AMTS, CDT, and MMSE and the IADL total score was computed to determine the latter’s sensitivity. The inter-rater reliability was computed using Cohen’s kappa coefficient (κ) for all IADL scale items. Finally, statistical significance was set at *p* < 0.05, and all reported *p*-values were two-tailed. The analysis was conducted using SPSS statistical software (version 19.0; Armonk, NY, USA).

## 3. Results

### 3.1. Demographic Data of the Samples

All groups were matched in age, gender, years of education, and marital status. The PD and PDD groups did not differ in the Hoehn–Yahr staging, while all patients were matched in the duration of disease (Table 1).

### 3.2. Comparison of Means between Subgroups

For a comparison of the IADL total mean score and its eight domain mean scores between the CHA group and the CIG, Student’s *t*-test was used. A statistically significant difference was observed in the IADL total score (*t*(106) = −9.26, *p* < 0.001), with the CHA group scoring significantly higher than the CIG. Likewise, statistically significant differences were observed in the eight domains of the IADL scale between the two groups: (a) “ability to use the telephone” (*t*(106) = −4.92, *p* < 0.001), (b) “shopping” (*t*(106) = −9.08, *p* < 0.001), (c) “food preparation” (*t*(106) = −8.00, *p* < 0.001), (d) “housekeeping” (*t*(106) = −5.17, *p* < 0.001), (e) “laundry” (*t*(106) = −6.21, *p* < 0.001), (f) “mode of transportation” (*t*(106) = −6.49, *p* < 0.001), (g) “responsibility for own medications” (*t*(106) = −6.22, *p* < 0.001), and (h) “ability to handle finances” (*t*(106) = −4.18, *p* < 0.001). According to these results, the CIG had lower scores in all IADL domains and in its total score (Table 2).

One-way ANOVA was used to compare the IADL total mean score and its eight domain mean scores between the CHA groups and the three clinical subgroups. A significant main effect of the group was found for the IADL total score (F(3, 128) = 34.07, *p* < 0.001, ηp^2^ = 0.63; Table 3), with AD patients having the lowest scores.

Likewise, statistically significant differences were observed in the eight domains of the IADL scale: (a) “ability to use the telephone” (*F*(3, 128) = 11.18, *p* < 0.001), (b) “shopping” (*F*(3, 128) = 28.56, *p* < 0.001), (c) “food preparation” (*F*(3, 128) = 22.62, *p* < 0.001), (d) “housekeeping” (*F*(3, 128) = 12.45, *p* < 0.001), (e) “laundry” (*F*(3, 128) = 13.54, *p* < 0.001), (f) “mode of transportation” (*F*(3, 128) = 21.22, *p* < 0.001), (g) “responsibility for own medications” (*F*(3, 128) = 12.27, *p* < 0.001), and (h) “ability to handle finances” (*F*(3, 128) = 6.76, *p* < 0.001). According to the analysis, the CIG obtained lower scores in all IADL items (Table 3).

Also, one-way ANOVA was used to explore the existence of the main effect of the group (the CHA group, the PD group, the PDD group, and the AD group) on the ABCD, AMTS, IADL, MMSE, and CDT total scores. The analysis showed a main effect of the group on all measures, specifically on the ABCD total scores (*F*(3, 128) = 63.73, *p* < 0.001; η_p_^2^ = 0.66), for the AMTS total score (*F*(3, 128) = 63.91, *p* < 0.001; η_p_^2^ = 0.55), for the CDT total score (*F*(3, 128) = 25.12, *p* < 0.001; η_p_^2^ = 0.66), and the MMSE total score (*F*(3, 128) = 41.20, *p* < 0.001; η_p_^2^ = 0.57). In all measurements, the AD and PDD patients obtained the lowest scores (see Table 4).

### 3.3. Receiver Operating Characteristic Analysis for the IADL Scale

**Figure 1:**
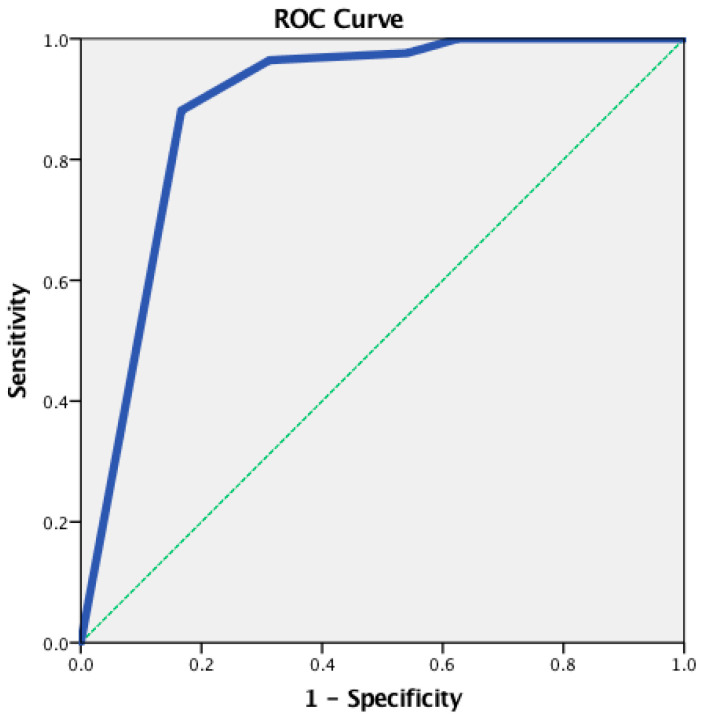
The receiver operating characteristic (ROC) curve for the IADL total score—between the CUG and the CIG.

An ROC analysis was conducted to determine the cut-off points for the IADL total score. A statistically significant positive discrimination between the CUG and the CIG was revealed (*AUC* = 0.888, *p* < 0.001). The cut-off point was equal to 6.00, with a sensitivity of 0.976 and a 1-specificity of 0.542 (Figure 1).

Additionally, the ROC analysis revealed a statistically significant positive discrimination between: (a) the CUG and the PDD group (*AUC* = 0.844 (95% CI: 0.795–0.983), *p* < 0.001), with the cut-off point being calculated at 6.00 (sensitivity 0.964 and 1-specificity 0.028); (b) the CUG and the AD group (*AUC* = 0.886 (95% CI: 0.792–0.980), *p* < 0.001), with the cut-off point being calculated at 6.00 (sensitivity 0.964 and 1-specificity 0.333); (c) the PD group and the PDD group (*AUC* = 0.820 (95% CI: 0.698–0.943), *p* < 0.001), with the cut-off point being calculated at 6.00 (sensitivity 0.875 and 1-specificity 0.292); (d) the PD group and the AD group (*AUC* = 0.810 (95% CI: 0.685–0.935), *p* < 0.001), with the cut-off point being calculated at 6.00 (sensitivity 0.875 and 1-specificity 0.333); (e) the CHA group and the PDD group (*AUC* = 0.917 (95% CI: 0.827–1.000), *p* < 0.001), with the cut-off point being calculated at 6.00 (sensitivity 1.000 and 1-specificity 0.292); and (f) the CHA group and the AD group (*AUC* = 0.917 (95% CI: 0.927–1.000), *p* < 0.001), with the cut-off point being calculated at 6.00 (sensitivity 1.000 and 1-specificity 0.333).

### 3.4. Reliability and Validity Measures for the IADL Scale

The overall estimated internal consistency of the IADL scale was excellent (Cronbach’s alpha = 0.890). Alternative analysis using the split-half reliability technique also showed that the IADL scale is internally consistent (split-half reliability coefficient = 0.901). The item scale correlations of the eight domains of the IADL scale are presented in Table 5.

### 3.5. Inter-Rater Reliability of the IADL Scale

The inter-rater agreement for the eight domains of Lawton’s IADL scale was computed using Cohen’s kappa coefficient (see Table 6).

### 3.6. Correlations for the IADL Scale

Pearson correlations were computed between the total scores of all the assessment tools used in this study and the IADL total scores to determine the latter’s sensitivity. The analysis used positive correlations between the IADL scale and the MMSE (*r* = 0.764, *p* < 0.001), the IADL scale and the AMTS (*r* = 0.769, *p* < 0.001), and the IADL scale and the ABCD (*r* = 0.702, *p* < 0.001), while a negative correlation was detected between the IADL scale and the CDT (*r* = 0.627, *p* < 0.001).

## 4. Discussion

This study presented the validation of the IADL measure in the Greek language for adults with cognitive impairment due to AD and PD. Additionally, the discriminatory value of the instrument was examined by means of an ROC analysis. In its current form, the Greek version of the IADL scale is proven valid and reliable as it provides robust results when assessing the functional abilities of individuals with cognitive impairment. Moreover, this unidimensional instrument proves to be a useful screener for clinicians for the assessment of the functional abilities of patients with cognitive impairment.

Specifically, it should be stressed that the Greek version of the IADL scale exhibits psychometric properties similar to those reported by other studies (Cronbach’s alpha: 0.830–0.968) [4,17,18,21,22,23,24,26]. Particularly, the internal consistency of the Greek IADL scale was excellent, approximately the same as the initial validation of the test [12], and similar to that of other linguistic and cultural adaptations of the IADL scale [4,16,18,19,20,26]. The psychometric scores calculated in this study are similar to the scores presented by the study that first attempted to offer preliminary results on the validation of the Greek version of the IADL scale (Cronbach’s alpha = 0.840) [25], to the results of a study on female advanced cancer patients in Greece (Cronbach’s alpha = 0.830) [26], and to a number of studies on European versions of the IADL scale [4,18]. The intraclass correlation coefficient for the Greek version of the IADL scale was excellent, a finding that agrees with previous validation studies [4,16,22,23,26] and that again reflects the excellent psychometric properties of the scale. Finally, results on the inter-rater agreements of the IADL scale were good, in line with results reported by other non-European validations of the measure [19,20,21,22,24].

Furthermore, the Greek version of the IADL scale validated with cognitively impaired adults showed good discriminant validity since it distinguished effectively cognitively healthy adults from those with cognitive impairment, with the former obtaining higher mean scores [20,25]. These findings underline the fact that the IADL measure can detect the changes occurring in the daily living of patients with cognitive impairment. Additionally, the Greek version of the IADL scale showed significant differences between the pre- and post-treatment of patients with cancer [26] and in elderly population studies [4,17,18,19,45]. This finding again demonstrates the discriminatory power of the IADL measure, especially in the case of Greek patients experiencing cognitive problems. Moreover, the results of the ROC analysis indicated that the Greek version of the IADL scale has discriminant validity with regard to the functional status between cognitively healthy individuals and those having different levels of impaired cognitive function [17,23,46,47], a finding that is in line with results obtained for other ADL screeners and in other studies [45,48]. The estimated threshold of 6.00 between the CUG and the CIG estimated for the IADL scale in this study is approximately the same as the cut-off provided in the study by Mystakidou et al. (2013) for advanced cancer female patients [26].

In terms of how the Greek version of the IADL scale correlates to other instruments testing cognitive impairment, a strong correlation was found, a finding that agrees with data from previous studies on Lawton’s IADL scale [12,17,23,25]. Specifically, a significant correlation between the IADL scale and the MMSE was reported in the study by Chin et al. (2018) on Korean patients with various neurodegenerative diseases (r = −0.646, *p* < 0.001) [23]. Moreover, Mathuranath et al.’s (2005) [17] study on older adults reported a good correlation between the IADL scale and the MMSE, a test that measures the extent of dementia and the level of cognitive impairment (r = −0.382, *p* = 0.009). The same strong significant correlation between the IADL scale and the MMSE was observed by Theotoka et al. (2007) in a study conducted on Alzheimer’s disease patients (r = 0.770, *p* < 0.001) [25]. In this study, the IADL scale correlated with the MMSE, CDT, AMTS, and ABCD, and all correlations were statistically significant (from 0.627 to 0.764), with the MMSE having a stronger correlation. Similar correlations were also observed in other validation studies on the IADL scale in which different factors and screening tests were correlated [4,20,21].

The above-mentioned results reveal that the Greek version of the IADL scale can detect changes in the daily living of patients with cognitive impairment due to AD and PD. This finding was also attested to in a previous study that stressed the importance of the IADL scale in identifying patients at risk of developing dementia [48], such as patients with mild cognitive impairment [49,50]. Furthermore, assessing a person’s level of independence and collecting information about their functional ability could help health professionals provide better treatment [4,51]. The IADL measure can be a valuable instrument to this end, along with the parallel use of other assessment tools [4,24]. Moreover, functional impairment is one of the core symptoms of AD [52]. Therefore, the IADL measure could provide important guidance to primary care physicians in diagnosing AD in the early stages [52]. In this line of thought, if the Greek version of the IADL is scale included in the screening process, it will expand the abilities of primary care physicians with regard to the early diagnosis of AD.

According to the literature, these changes in patients’ daily living could affect their quality of life [4,5]. Specifically, factors influencing the patients’ QoL include depression and functional impairment. However, proxy reports are necessary to assess all perspectives of the QoL, indicating that cognitive and functional impairments and neuropsychiatric symptoms significantly impact patients’ QoL [53].

### Strengths and Limitations

One limitation of this study is its small sample size. Therefore, the experiment should be replicated with larger samples including participants with different types of dementia (e.g., frontotemporal), which could improve the possibility of generalization of the results reported in this study. However, it should be noted that this sample was clearly classified using most of the criteria proposed by the NIA–AA [6,7,8] and the MDS task force [27,28,29,30]. Additionally, another strength of this study is that to the best of our knowledge, this is the first study that included individuals with dementia of different etiologies.

## 5. Conclusions

The results of this study confirm the validity of Lawton’s IADL scale as a reliable tool for assessing Greek-speaking adults with cognitive impairment due to AD and PD. This instrument demonstrated good reliability and validity based on the obtained data. Additionally, the IADL scale presented screening properties common to other ADL tools. Furthermore, Lawton’s IADL scale can profoundly distinguish between “cognitively healthy individuals” and those with “cognitive impairment”. All results reported in this study are in accordance with relevant results obtained by other IADL scale cultural and language adaptation studies. Therefore, this study offered a solid start for validating Lawton’s IADL scale and provided solid results on the usefulness of the measure in the screening of an individual’s cognitive abilities. In addition, it would be worthwhile if advanced IADL-based research is carried out with patients of different cognitive staging. Such research could provide predictive information regarding daily life activities related to cognitive problems.

Finally, another fruitful line of investigation would be research conducted with other populations that face difficulties in their daily living, as in Mystakidou’s study on cancer patients [26]. Finally, the use of the IADL measure as an important screening tool in first-degree healthcare settings in Greece as well as in other European countries is worth considering. Lawton’s IADL cut-off score, combined with other cognitive assessment tools in daily clinical settings, could help clinicians’ better profile the cognitively impaired populations they are working with. In the long run, this could help clinicians better monitor their patients by comparing the early and late effects of cognitive impairment in terms of Lawton’s IADL scores and cut-off points. This, consequently, can lead to more customized treatment planning.

## Figures and Tables

**Table 1:** Demographic characteristics of the samples.

	CHA Group N = 60	PD Patients N = 24	PDD Patients N = 24	AD Patients N = 24	*p*-Value
	M (SD)	M (SD)	M (SD)	M (SD)	
Years of age	67.77 (7.51)	69.00 (5.81)	71.54 (9.15)	67.58 (7.11)	0.446 ^†^
Gender, N (%)					
Male	30 (50%)	12 (50%)	13 (54%)	13 (54%)	
Female	30 (50%)	12 (50%)	11 (46%)	11 (46%)	0.575 ^‡^
Family status					
Married, N (%)	60 (100%)	24 (100%)	14 (100%)	24 (100%)	
Years of education	8.07 (3.86)	9.23 (4.89)	8.35 (3.55)	9.54 (3.68)	0.222 ^†^
Duration of disease	------	2–3 years	2–3 years	2–3 years	
H–Y staging	------	1. 64 (0.056)	1.59 (0.038)	------	0.741 ^†^

Abbreviations: CHA, cognitive healthy adult; PD, Parkinson’s disease; PDD, Parkinson’s disease dementia; AD, Alzheimer’s disease; H–Y staging, Hoehn–Yahr staging; ^†^ one-way ANOVA; ^‡^ Pearson’s χ^2^ test.

**Table 2:** Cognitive healthy adult group and cognitive impaired group comparisons in the IADL total mean score and eight domain mean scores.

	CIG Group (N = 48)	CHA Group (N = 60)		
	Μ (SD)	Μ (SD)	*t*(106)	*p*-Value
Ability to use the telephone	0.71 (0.46)	1.00 (0.00)	−4.92	<0.001
Shopping	0.42 (0.49)	1.00 (0.00)	−9.08	<0.001
Food preparation	0.48 (0.50)	1.00 (0.00)	−8.00	<0.001
Housekeeping	0.68 (0.46)	1.00 (0.00)	−5.17	<0.001
Laundry	0.61 (0.49)	1.00 (0.00)	−6.21	<0.001
Mode of transportation	0.58 (0.49)	1.00 (0.00)	−6.49	<0.001
Responsibility for own medications	0.60 (0.60)	1.00 (0.00)	−6.22	<0.001
Ability to handle finances	0.77 (0.42)	1.00 (0.00)	−4.18	<0.001
IADL total score	4.85 (2.63)	8.00 (0.00)	−9.26	<0.001

Abbreviations: CHA, cognitive healthy adult; CIG, cognitive impaired group; SD, standard deviation; IADL, Instrumental Abilities of Daily Living.

**Table 3:** Group effects (CHA, PD, PDD, AD) on the IADL total mean score and eight domain mean scores.

	CHA Group N = 60	PD Patients N = 24	PDD Patients N = 24	AD Patients N = 24		
	Μ (SD)	Μ (SD)	Μ (SD)	Μ (SD)	F (3, 128)	*p*-Value
Ability to use the telephone	1.00 (0.00)	1.00 (0.00)	0.71 (0.46)	0.70 (0.46)	11.18	<0.001
Shopping	1.00 (0.00)	0.88 (0.33)	0.46 (0.50)	0.38 (0.49)	28.56	<0.001
Food preparation	1.00 (0.00)	0.88 (0.33)	0.54 (0.50)	0.42 (0.50)	22.62	<0.001
Housekeeping	1.00 (0.00)	1.00 (0.00)	0.71 (0.46)	0.67 (0.48)	12.45	<0.001
Laundry	1.00 (0.00)	0.88 (0.33)	0.66 (0.48)	0.54 (0.50)	13.54	<0.001
Mode of transportation	1.00 (0.00)	1.00 (0.00)	0.50 (0.51)	0.66 (0.48)	21.22	<0.001
Responsibility for own medications	1.00 (0.00)	0.79 (0.41)	0.54 (0.50)	0.67 (0.48)	12.27	<0.001
Ability to handle finances	1.00 (0.00)	0.95 (0.20)	0.79 (0.41)	0.75 (0.44)	6.76	<0.001
IADL total score	8.00 (0.00)	7.37 (0.92)	4.91 (2.46)	4.79 (2.84)	34.07	<0.001

Abbreviations: CHA, cognitive healthy adult; PD, Parkinson’s disease; PDD, Parkinson’s disease dementia; AD, Alzheimer’s disease; SD, standard deviation; IADL, Instrumental Abilities of Daily Living.

**Table 4:** Group effects (CHA, PD, PDD, AD) on the ABCD, AMTS, IADL, MMSE, and CDT total mean scores.

	CHA Group N = 60	PD Patients N = 24	PDD Patients N = 24	AD Patients N = 24		
	Μ (SD)	Μ (SD)	Μ (SD)	Μ (SD)	F (3, 128)	η^2^
ABCD	21.53 (1.35)	22.10 (1.70)	16.18 (4.54)	16.67 (2.76)	63.73 *	0.66
AMTS	9.60 (0.55)	9.06 (1.10)	6.05 (1.03)	6.38 (1.38)	63.919 *	0.55
MMSE	29.47 (0.65)	28.00 (1.13)	21.21 (4.13)	19.29 (6.74)	41.20 *	0.57
CDT	6.54 (0.24)	6.35 (1.01)	2.96 (2.62)	3.17 (2.94)	25.12 *	0.66

Abbreviations: CHA, cognitive healthy adult; PD, Parkinson disease; PDD, Parkinson disease dementia; AD, Alzheimer disease; SD, standard deviation; AMTS, Abbreviated Mental Test Score; MMSE, Mini Mental State Examination; ABCD, Arizona Battery for Communication Disorders of Dementia; * p < 0.001.

**Table 5:** Item scale correlations and reliability measures of the IADL domains.

IADL Domains	Item Scale Correlation
Ability to use the telephone	0.869
Shopping	0.879
Food preparation	0.873
Housekeeping	0.876
Laundry	0.887
Mode of transportation	0.870
Responsibility for own medications	0.875
Ability to handle finances	0.881

**Table 6:** Inter-rater agreement of the IADL domains for the cognitively impaired group (CIG).

IADL Domains	*κ*	*p*-Value
Ability to use the telephone	0.948	<0.001
Shopping	0.832	<0.001
Food preparation	0.839	<0.001
Housekeeping	0.874	<0.001
Laundry	0.957	<0.001
Mode of transportation	0.958	<0.001
Responsibility for own medications	1.000	<0.001
Ability to handle finances	0.845	<0.001

## Data Availability

The data are unavailable due to privacy or ethical restrictions.
